# A Study on the Bidirectional Effects of Depression and Household Savings: Empirical Evidence from CHARLS

**DOI:** 10.3390/healthcare14101397

**Published:** 2026-05-20

**Authors:** Yan Wang, Ruxin Kou, Qianqian Xu, Yuanyang Wu, Haixia Wang, Xinping Zhang

**Affiliations:** School of Medicine and Health Management, Tongji Medical College, Huazhong University of Science and Technology, Wuhan 430030, China; m202576110@hust.edu.cn (Y.W.); kouruxin@163.com (R.K.); qianqian93xu93@163.com (Q.X.); wuyuanyang97225@163.com (Y.W.); m202476082@hust.edu.cn (H.W.)

**Keywords:** depression, savings, middle-aged and senior people, two-way relationship, cross-lag panel model

## Abstract

**Background**: Depression has become a prevalent and serious public health problem worldwide, attracting widespread attention from governments and international organizations and being included in key health policy issues. There is a close interaction between depression and economic factors such as household savings, a link particularly pronounced in Asian countries. However, evidence of a two-way association is limited. This study aims to explore the two-way relationship between depression and savings among middle-aged and older adults. **Methods**: Data were collected from 6746 respondents in the China Health and Retirement Longitudinal Study (CHARLS) in 2013, 2015, and 2018. A cross-lagged panel model was used to test the two-way relationship between depression and savings. **Results**: The results showed a significant cross-time effect of depression on savings (T0 → T1: β = −0.052; T1 → T2: β = −0.077), suggesting that higher levels of depression in the early stages are associated with lower levels of savings later. Moreover, savings had a stronger negative predictive effect on depression (T0 → T1: β = −0.463; T1 → T2: β = −0.510), indicating that higher levels of savings in the early stages are associated with lower levels of depression later. Furthermore, the grouping test showed that the two-way negative effect remained stable in both male and female groups (*p* ≤ 0.001). **Conclusions**: This study reveals the dynamic two-way influence between depression and savings, providing a basis for policy formulation that synergistically promotes residents’ mental health and household financial stability.

## 1. Introduction

More than one billion people worldwide suffer from mental health disorders, with anxiety and depression being the most common [1]. Over the past fifteen years, the prevalence of depression has continued to rise [2,3]. According to data from the U.S. National Institute of Mental Health, more than 21 million adults experienced at least one major depression in the past year, and the proportion of adults reporting increased anxiety symptoms ranges from 6% to 44% [4,5,6]. And the global burden of depression continues to increase, with the combined costs of related health care and productivity loss estimated to exceed US$326 billion [6,7]. Moreover, approximately 60% of suicide cases are associated with mental disorders such as depression [8]. Preventing and alleviating depression and improving mental health have therefore become central topics in global health strategies and national policy agendas.

A growing body of research has explored depression from an economic perspective, focusing largely on macro- or meso-level indicators such as income, debt, and financial stress. Existing studies have confirmed that financial hardship, including low income and accumulated debt, significantly increases the risk of depression, whereas improved economic conditions may have a protective effect [9]. But the savings reflecting household economic conditions and capacity for resource accumulation may also affect mental health, yet the evidence remains relatively limited. In many studies, mental health is included merely as a control variable when analyzing savings behavior [10]. Most existing work assumes a one-way effect of mental health on savings and overlooks the possible reverse effect of household financial conditions on mental health [11]. Whether a bidirectional relationship exists and what mechanisms exist remain insufficiently understood.

Globally, the share of the population aged 65 and above continues to rise. The population aged 60 and above had reached 310 million, including 220 million people aged 65 and above in China by the end of 2024 [12]. As population aging accelerates, social competition intensifies, and uncertainties associated with economic transition increase, the internal linkage between the depression of middle-aged & older adults and savings has become more prominent. And this topic has emerged as an important research direction at the intersection of sociology, economics, and public health. So, exploring depression among Chinese middle-aged and older adults characterized as high-saving is both urgent and necessary [13].

Depression refers to a common mental disorder characterized by persistent low mood, loss of interest or pleasure, and related emotional and behavioral symptoms lasting for an extended period [14]. Household savings in this study refer to the total amount of money deposited by respondents in formal financial institutions such as banks, which reflects the household’s accumulated financial resources and liquidity buffer [15].

Against this background, this study uses data from the China Health and Retirement Longitudinal Study (CHARLS) to construct a cross-lagged panel model. The primary research question addressed in this study is: What is the bidirectional longitudinal relationship between depressive symptoms and household savings among middle-aged and older adults in China, and do these two factors predict each other over time? Specifically, we test whether depression and savings exhibit a significant bidirectional negative relationship, while separating between-person differences from within-person dynamic changes to identify lagged effects more precisely. The findings are expected to offer methodological guidance for research on similar bidirectional relationships [16].

## 2. Materials and Methods

### 2.1. Study Design

This study is a longitudinal observational study based on three waves of national representative survey data. A cross-lagged panel model (CLPM) was used to examine the directionality and temporal dynamics of the bidirectional relationship between depressive symptoms and household savings across 2013, 2015, and 2018.

### 2.2. Data Sources

The data for this study were sourced from the China Health and Retire Longitudinal Study (CHARLS), organized by the National School of Development at Peking University. CHARLS aims to collect high-quality micro-level data representing households and individuals aged 45 and above in China to analyze the country’s aging population dynamics and advance interdisciplinary research on aging issues. The study employed a multi-stage stratified probability sampling design to ensure national sample representativeness, covering 150 counties/districts and 450 villages/township communities across 28 provinces nationwide. CHARLS is an ongoing survey project conducted every 2 to 3 years. All interviews were conducted by trained researchers using standardized questionnaires [17].

**Figure 1:**
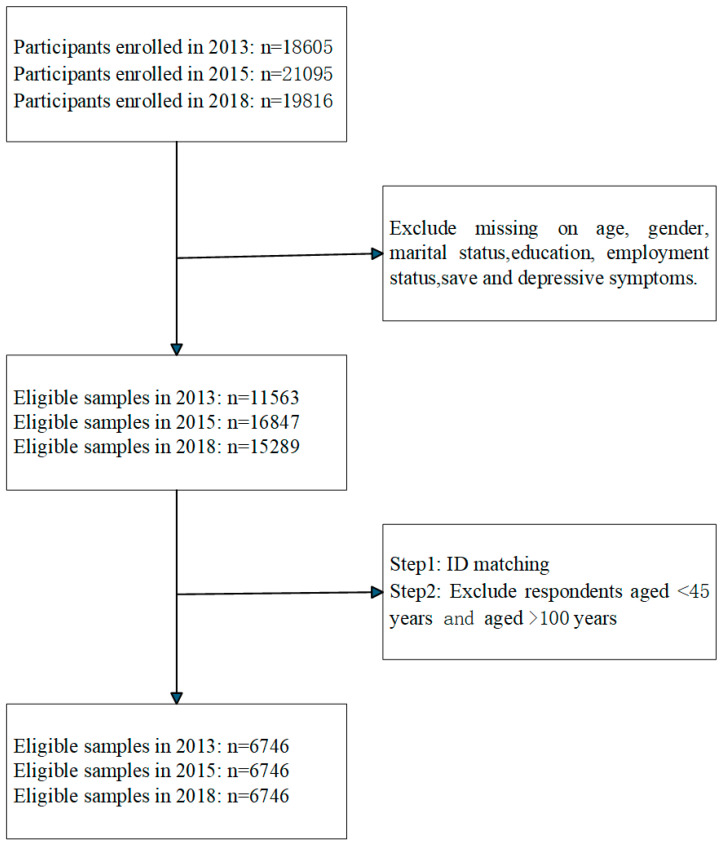
Flow chart for participant inclusion and exclusion.

The data used in this study were drawn from the 2013, 2015, and 2018 waves of the CHARLS [18,19]. The inclusion criteria were as follows: (1) respondents aged 45 years or above and younger than 100 years; and (2) no missing values in core variables, such as depression and savings. A final sample of 6746 respondents was included. See Figure 1 for details.

### 2.3. Measurement of Depression

Depression was measured using the 10-item Center for Epidemiologic Studies Depression Scale (CES-D-10) [20,21]. It contains 10 items scored on a four-point scale: “rarely or none of the time (less than 1 day),” “some or a little of the time (1–2 days),” “occasionally or a moderate amount of the time (3–4 days),” and “most or all of the time (5–7 days),” coded as 0, 1, 2, and 3, respectively. Items 5 and 8 are reverse-coded. Total scores range from 0 to 30, and a score of 10 or above was defined as indicating depressive symptoms (depression in short).

### 2.4. Measurement of Savings

Savings were measured by the survey question: “How much money do you currently have deposited in financial institutions (such as banks)?” This indicator was chosen because it is a widely used, objective, and comparable measure of formal household savings in large-scale longitudinal surveys. It reflects readily available liquid financial resources and has high reliability and low reporting bias compared with other subjective financial well-being indicators. Because the distribution of savings is typically highly right-skewed in the population, it was transformed using the natural logarithm.

### 2.5. Control Variables

To eliminate interference from other factors, a set of control variables was designed in the analysis. These variables were age, gender, marital status (distinguishing between married and other statuses), educational attainment (classified by level of academic qualification), and employment status (employed and unemployed).

### 2.6. Statistical Analysis

Descriptive statistics were produced using IBM SPSS Statistics 27. Spearman correlation analysis and cross-lagged analysis were performed in Stata 18 to assess the relationship between depression and savings. Longitudinal changes across the three time points were examined using a cross-lagged panel model (CLPM) to test their bidirectional relationship [22,23]. 

## 3. Results

### 3.1. Descriptive Analyses

Demographically, the depression group had significantly higher proportions of individuals aged 56–65 compared to the non-depression group (*p* < 0.05). In 2018, the depression group had a significantly higher proportion of individuals aged 45–55. The depression group consistently had higher proportions in the 66–75 age group across all waves (*p* < 0.05). Moreover, women exhibited a significantly higher prevalence of depression across all three waves. The proportion of the employed was significantly higher in the depression. The depression group exhibited a significantly higher proportion of individuals with a secondary education or higher in 2013, but this proportion declined by 2018. Regarding marital status, the depression group consistently showed a significantly higher proportion of married individuals across all survey cycles; contrary to the initial description, the proportion of widowed or divorced individuals in this group was generally lower. Detailed information is presented in Table 1.

The depression and non-depression groups showed significant differences in savings levels and key demographic characteristics. Independent-samples *t*-tests were used to compare savings levels between groups. The savings of the non-depression consistently exceeded those of the depression at *p* < 0.001. Taking 2013 as an example, the non-depression’s savings were 8.97, 0.60 higher than the depression’s 8.37. Significant differences were also found between the two groups in age distribution, gender composition, work status, education level, and marital status (all *p* < 0.05).

### 3.2. Correlation Analyses

Savings and depression showed significant negative correlations at all time points < 0.05). Each correlation analysis included only samples where both depressive symptoms and savings behavior variables were complete at the corresponding time points. Consequently, the effective sample sizes for the three analyses were 2194, 2145, and 2465, respectively, all representing subsamples from the total matched sample of 6746 participants. The Pearson correlation coefficient was −0.139 at T0, −0.159 at T1, −0.204 at T2 (Table 2). Detailed results, including the correlation coefficient (r), sample size (n), degrees of freedom (df = n − 2), *p*-value, and 95% confidence interval (95% CI) for each wave, are presented in Table 3. The negative correlation relationship remained consistently stable over the three-year period, indicating a consistent negative association between savings and depression during the same timeframe.

### 3.3. The Cross-Lagged Model

In summary, this cross-lagged analysis clearly delineates the dynamic relationship pattern between depression and savings. It not only confirms the temporal stability of both variables but also reveals their mutually inhibitory cross-temporal effects, providing empirical evidence for understanding the longitudinal interactive mechanism between depression and savings.

The CLPM demonstrated excellent fit (CFI = 0.965, TLI = 0.958, SRMR = 0.042, RMSEA = 0.038), with all fit indices meeting acceptable standards.

The autoregressive effects between depression and savings indicate that both depression and savings exhibit significant self-stability, with earlier states effectively predicting later states. The coefficient for depression reached 0.624 from T0 to T1 and 0.557 from T1 to T2. The effect for savings is also significant, with coefficients of 0.404 from T0 to T1 and 0.507 from T1 to T2. Complete CLPM results are presented in Table 4.

Second, depression at point T0 exerted a significant negative effect on savings at point T1 (β = −0.052). while depression at T1 negatively predicted savings at T2 (β = −0.077). Regarding the cross-lagged effect of savings on depression: savings at T0 exhibited a negative coefficient of −0.463 on depression at T1, while the negative coefficient for savings at T1 on depression at T2 further increased to −0.510. Detailed results are shown in Table 4.

Finally, the residual correlation results reveal the simultaneous association between the two variables at the same time point. After controlling for the effects of autoregressive and cross-lagged paths, depression and savings still exhibit significant negative residual correlations at points T0, T1, and T2, with coefficients of −0.057, −0.049, and −0.081.

### 3.4. Robustness Test

#### 3.4.1. Subsample Analysis—CLPM

**Figure 2:**
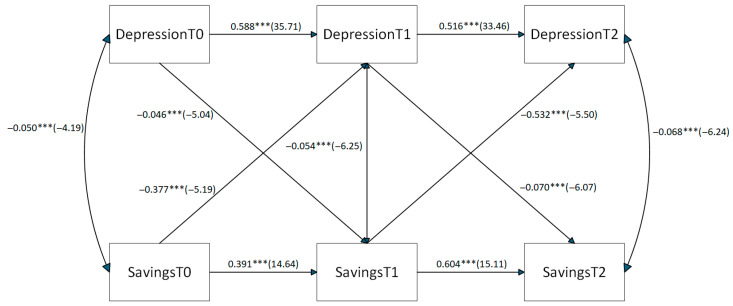
Path Diagram of Depression and Savings CLPM for Males. The significance level in the table is marked as: *** *p* ≤ 0.001.

**Figure 3:**
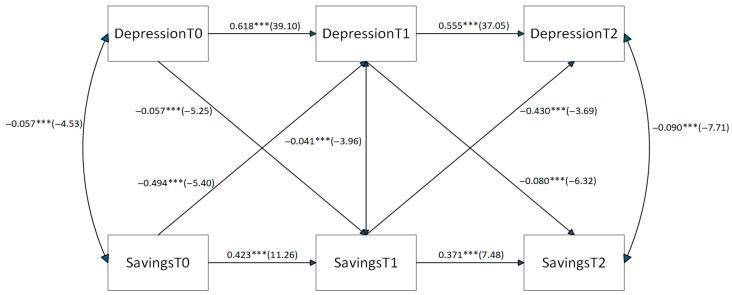
Path Diagram of Depression and Savings CLPM for Females. The significance level in the table is marked as: *** *p* ≤ 0.001.

To validate the robustness of the bidirectional relationship between depression and savings derived from CHARLS data from 2013, 2015, and 2018, this study employs a gender-subsample testing strategy. The CLPM path diagram for the male subsample is shown in Figure 2. The core bidirectional cross-lagged paths are significant and consistent in direction with the full sample: The path coefficients for 2013 depression → 2015 savings and 2013 savings → 2015 depression were −0.046 (−5.04) and −0.377 (−5.19), respectively, indicating negative effects. Subsequent cross-lagged paths from 2015 to 2018 maintained this characteristic. The CLPM path diagram for the female subsample, as shown in Figure 3, exhibits both commonalities and distinct differences with the male group. The commonality lies in the significant positive autoregressive path between depression and savings, with the path coefficient for 2013 depression → 2015 savings being −0.057 (−5.25), indicating a significant negative effect with an intensity close to that observed in males. The difference lies in the path coefficient for the 2013 savings → 2015 depression path in the female group, which is −0.494 (−5.40), significantly larger than that for males. This indicates that female savings behavior exhibits stronger temporal stability.

Comparing the core characteristics of the two path diagrams reveals that the conclusions of this study exhibit reliable robustness. The bidirectional negative impact pathway between depression and savings holds significantly for both male and female groups, fully consistent with the core features of the CLPM path diagram for the entire sample.

#### 3.4.2. Robustness Analysis: Stepwise Nested Model Testing

Table 5 presents the robustness test results for the relationship between depression and savings. Regarding model specification robustness, this study employs a stepwise nested model strategy: The baseline model (Model 1) includes only one independent variable—savings amount—which exhibits a significant negative effect on depression (e.g., β = −0.345 in 2013). In model 2, which adds the control variables age and gender, the coefficient is −0.314; In model 3, which adds all control variables, the coefficient is −0.291. This result enhanced the explanatory power of the core association; it further corroborated the model’s validity and the robustness of the core conclusion [23].

In terms of robustness across time dimensions, the results from 2013, 2015, and 2018 demonstrate high consistency: the correlation coefficient between the core variable and depression was β = −0.291 in 2013, β = −0.460 in 2015, and β = −0.465 in 2018. Not only did the coefficients maintain a negative direction across all three time points, but they also passed the statistical significance test at *p* < 0.001. This cross-year stability of results rules out the possibility that the association between depression and savings is a random fluctuation specific to a particular time point.

## 4. Discussion

This study found that there is a significant two-way negative relationship between depression and household savings in the middle-aged and elderly population. Specifically, the more severe the depression in the early stage, the lower the savings in the later stage; at the same time, the higher the savings in the early stage, the lower subsequent depression and its aggravation. This conclusion breaks through the limitations of previous studies that focused on one-way causal relationships and systematically confirms the existence of a dynamic two-way circulation mechanism between the two in the middle-aged and elderly population [9].

This two-way circulation mechanism can be realized through specific behavioral paths. Depressive symptoms can significantly inhibit the accumulation process of household savings by reducing individual labor participation rate, weakening risk-taking willingness, and distorting consumption decision logic [24]; while insufficient savings will further weaken the family’s financial buffer capacity to cope with emergencies, exacerbate the individual’s economic insecurity and sense of loss of control over life, and thus induce or aggravate depression [24,25]. The results of the study confirm the role of savings in depression. This finding supports the previous evidence that the higher the personal income, the lower the probability of clinically meaningful depression reports [25]. Furthermore, the findings of this study also confirm that when people’s financial situation improves, their stress is reduced, which in turn reduces the occurrence of stress-related conditions such as depression [25,26].

Depression was more prevalent among older adults and women. These groups typically face greater financial strain, weaker social support, and fewer coping resources, which increase their vulnerability to both economic disadvantage and depressive symptoms. The baseline disparities observed in Table 1 help explain the observed longitudinal bidirectional relationship.

Compared with existing studies, the core contribution of this study is to break through the limitations of one-way influence analysis and systematically confirm the dynamic two-way cyclical relationship between depression and savings in the middle-aged and elderly population. Although some studies have found that unemployment and reduced family income are closely related to the occurrence of mood disorders [27], the above studies are mostly focused on the one-way association between income indicators and mental health. This study further reveals the cyclical mechanism that depression inhibits savings through income and risk preference pathways, and insufficient savings exacerbate depression in turn by weakening economic security. This is consistent with the pattern of “health and behavior forming a cycle” in the literature [28,29], but it is the first time to extend it to the field of mental health and family savings.

These results support the economic resource hypothesis, which states that sufficient financial reserves help alleviate liquidity constraints, strengthen medical and emergency preparedness, reduce anxiety and depressive symptoms, and thus improve subjective well-being [30]. This process is highly consistent with the core proposition of the resource conservation theory, which states that when individuals are in a state of resource depletion, they tend to adopt contractionary economic strategies to avoid potential losses. However, such short-term coping behaviors will exacerbate the vulnerability of the family economy in the long run, eventually forming a vicious cycle that is difficult to break [5]. This study provides new empirical evidence for the application of the resource conservation theory in the intersection of mental health and family finance and reveals the dynamic cycle mechanism of “resource depletion—contractionary behavior—increased vulnerability—worsening symptoms” between depressive symptoms and family savings [31], thus expanding the explanatory boundaries of the economic resource hypothesis. At the same time, this study verified the two-way causal relationship between the two through a cross-lag model, providing a methodological reference for understanding the dynamic linkage between mental health and economic behavior.

It is noteworthy that the mutual influence between the two varies slightly across different tracking periods, with a more pronounced short-term effect and a gradually diminishing long-term effect. This may indicate that the dynamic relationship between depression and savings is more sensitive in the short term, while in the long term, it may be influenced by other mediating factors or adaptive regulatory mechanisms.

Despite its contributions, the study has limitations. First, there are limitations at the data level. CHARLS relies on self-reported indicators to measure both household savings and depressive symptoms. Although the dataset is nationally representative, self-reported savings balances and saving rates may be measured with error, and the data cannot capture detailed uses of savings. Moreover, although the CES-D-10 is widely used to assess depressive symptoms, it cannot replace clinical diagnosis and therefore cannot fully distinguish the heterogeneous effects of depressive mood and depressive disorder. Second, there are methodological limitations. Although the cross-lagged model helps separate between-person differences from within-person changes and the findings are supported by subsample and robustness analyses, omitted-variable bias cannot be completely ruled out. Third, the analysis failed to adjust for key confounding variables, including chronic somatic diseases, neurological and neuropsychiatric conditions, as well as alcohol and tobacco use. These factors may simultaneously influence depressive symptoms and household economic behaviors, thereby introducing potential confounding bias into the estimated associations.

Future studies could use longer panel data and more comprehensive financial indicators to improve measurement accuracy. Additional confounders such as chronic diseases, alcohol and tobacco use should be included. Further research may also explore potential mediating and moderating factors in the bidirectional relationship. Policies should integrate mental health services with financial security support for middle-aged and older adults. Targeted interventions should be provided to vulnerable groups. Improving financial literacy and social security can help break the negative cycle between depression and insufficient savings.

## 5. Conclusions

This study utilized three waves of longitudinal data from the China Health and Retire Longitudinal Study (CHARLS) in 2013, 2015, and 2018, employing a cross-lag panel model to systematically examine the dynamic bidirectional relationship between depressive symptoms and household savings among middle-aged and elderly individuals aged 45 and above. The results demonstrated a significant and robust negative bidirectional relationship between depressive symptoms and household savings: more severe depressive symptoms at earlier time points were associated with significantly lower household savings levels later on, while higher household savings levels at earlier time points were linked to significantly milder depressive symptoms subsequently. This bidirectional effect remained consistent across both male and female subgroups and persisted after controlling for sociodemographic variables such as age, gender, marital status, employment, and education.

This study reveals that a mutually reinforcing dynamic cyclical mechanism exists between mental health and household financial security in middle-aged and elderly populations: depression may suppress household savings accumulation by reducing labor participation, impairing risk cognition, and weakening rational economic decision-making [24]; conversely, insufficient savings weaken household financial buffers and risk resilience, exacerbating economic insecurity and life stress, thereby inducing or worsening depression [24,25]. These findings provide longitudinal empirical support from the China context for the Resource Conservation Theory and the Economic Resource Hypothesis, demonstrating that the relationship between mental health and economic behavior is not unidirectional causal but rather a closed-loop dynamic interaction over the long term.

This study highlights the urgent need to integrate mental health services with financial protection policies: regular depression screening and psychological support should be provided to vulnerable groups such as the elderly, women, individuals with low education levels, and the unemployed; simultaneously, the social security system should be enhanced, residents’ financial literacy improved, and rational savings practices guided to strengthen family risk resilience. Breaking the vicious cycle between depression and insufficient savings will not only improve the mental health and financial well-being of middle-aged and elderly populations but also holds significant practical implications for advancing healthy aging and sustainable family development amid accelerating population aging.

## Figures and Tables

**Table 1:** Baseline Characteristics of the Study Population.

Variables	2013			2015			2018		
	Depression	Non-Depression	*p*	Depression	Non-Depression	*p*	Depression	Non-Depression	*p*
Savings, mean (SD)	8.372 (2.110)	8.969 (2.059)	0.001 ***	8.982 (1.772)	9.622 (1.611)	0.001 ***	8.645 (2.719)	9.489 (2.112)	0.001 ***
Age, N (%)	2070	4671		2229	4515		2604	4128	
45–55	659 (31.84)	1599 (34.23)	0.082	589 (26.43)	1261 (27.93)	0.006 ***	365 (14.02)	569 (13.78)	0.001 ***
56–65	914 (44.15)	1980 (42.39)	0.001 ***	913 (40.96)	1880 (41.64)	0.001 ***	1077 (41.36)	1720 (41.67)	0.001 ***
66–75	437 (21.11)	911 (19.50)	0.001 ***	624 (27.99)	1133 (25.10)	0.001 ***	901 (34.60)	1424 (34.50)	0.001 ***
76–85	58 (2.80)	177 (3.79)	0.008 ***	101 (4.53)	230 (5.09)	0.322	249 (9.56)	399 (9.67)	0.039 **
>86	2 (0.10)	4 (0.09)	-	2 (0.09)	11 (0.24)	-	12 (0.46)	16 (0.39)	0.999
Gender, N (%)	2071	4675		2230	4516		2610	4136	
Male	809 (39.06)	2562 (54.80)	0.001 ***	847 (37.98)	2524 (55.87)	0.001 ***	1036 (44.36)	2335 (69.42)	0.001 ***
Female	1262 (60.94)	2113 (45.20)	0.001 ***	1383 (62.02)	1992 (44.13)	0.001 ***	1574 (55.64)	1801 (30.58)	0.001 ***
Work Status, N (%)	2071	4675		2230	4516		2610	4136	
Employed	1381 (66.87)	2869 (61.23)	0.001 ***	855 (83.63)	2534 (56.92)	0.001 ***	1455 (61.72)	2162 (64.26)	0.001 ***
Unemployed	690 (33.13)	1806 (38.77)	0.001 ***	166 (16.37)	1982 (43.08)	0.001 ***	1155 (38.28)	1974 (35.74)	0.001 ***
Education level, N (%)	1649	3683		2228	4511		2610	4136	
No formal education	150 (7.22)	295 (6.29)	0.75	166 (16.27)	312 (6.99)	0.343	687 (29.33)	1064 (31.62)	0.001 ***
Primary school	125 (6.02)	279 (5.93)	0.001 ***	222 (21.75)	450 (10.09)	0.050 **	1140 (48.81)	1821 (54.09)	0.001 ***
Secondary school and above	1374 (86.76)	3109 (67.78)	0.001 ***	1840 (61.98)	3749 (82.92)	0.001 ***	783 (33.35)	1251 (37.20)	0.001 ***
Marital status, N (%)	2071	4672		2228	4514		2610	4136	
Married	1844 (92.42)	4100 (87.23)	0.001 ***	1950 (95.57)	3888 (87.27)	0.001 ***	2187 (93.28)	3417 (91.53)	0.001 ***
Unmarried	14 (0.70)	42 (0.89)	0.927	10 (0.49)	40 (0.90)	-	17 (0.72)	28 (0.75)	0.929
Divorced	12 (0.60)	35 (0.74)	0.49	12 (0.59)	32 (0.72)	0.329	28 (1.19)	29 (0.77)	0.063 *
Widowed	192 (9.62)	473 (10.03)	0.014	249 (12.21)	541 (12.15)	0.422	367 (15.69)	645 (17.20)	0.001 ***
Other	9 (0.45)	22 (0.47)	-	7 (0.34)	13 (0.29)	-	11 (0.47)	17 (0.45)	-

Note: Data are presented as N (%). Percentages are based on the total sample of each category and may not sum to 100% due to rounding. *** *p* ≤ 0.001, ** *p* ≤ 0.01, * *p* ≤ 0.05.

**Table 2:** Pearson Correlation Analysis Results for Depression and Savings (2013, 2015, 2018).

	Depression T0	Savings T0	Depression T1	Savings T1	Depression T2	Savings T2
Depression T0	1.000					
Savings T0	−0.139 *	1.000				
Depression T1	0.559 *	−0.168 *	1.000			
Savings T1	−0.162 *	0.469 *	−0.159 *	1.000		
Depression T2	0.508 *	−0.103 *	0.539 *	−0.145 *	1.000	
Savings T2	−0.188 *	0.334 *	−0.182 *	0.408 *	−0.204 *	1.000

Note: T0, T1, and T2 represent the three survey periods in this study (i.e., the 2013 baseline, the first follow-up in 2015, and the second follow-up in 2018). Specifically, Depression T0 denotes depression in 2013, while Savings T0 represents savings amounts in the same year. Depression T1 and Savings T1 denote observations from 2015, and Depression T2 and Savings T2 denote observations from 2018. The significance level in the table is marked as: * *p* ≤ 0.05.

**Table 3:** Pearson Correlation Coefficients Between Depression and Savings at Each Wave.

Wave	Variable Pair	r	N	df = n − 2	*p*-Value	95% CI
T0 (2013)	DepressionT0 & SavingsT0	−0.139	2194	2192	0.001 ***	[−0.162, −0.116]
T1 (2015)	DepressionT1 & SavingsT1	−0.159	2145	2143	0.001 ***	[−0.181, −0.099]
T2 (2018)	DepressionT2 & SavingsT2	−0.204	2465	2463	0.001 ***	[−0.200, −0.117]

The significance level in the table is marked as: *** *p* ≤ 0.001.

**Table 4:** Results of Cross-Lagged Analysis of Depression and Savings.

Path	*β*	SE	*t*
Auto-regressive paths			
DepressionT0 → DepressionT1	0.624 ***	0.011	55.36
DepressionT1 → DepressionT2	0.557 ***	0.011	52.57
SavingsT0 → SavingsT1	0.404 ***	0.022	18.44
SavingsT1 → SavingsT2	0.507 ***	0.031	16.25
Cross-lagged paths			
DepressionT0 → SavingsT1	−0.052 ***	0.007	−7.58
SavingsT0 → DepressionT1	−0.463 ***	0.058	−7.99
DepressionT1 → SavingsT2	−0.077 ***	0.008	−9.21
SavingsT1 → DepressionT2	−0.510 ***	0.075	−6.78
Residual correlations			
DepressionT0 → SavingsT0	−0.057 ***	0.009	−6.62
DepressionT1 → SavingsT1	−0.049 ***	0.007	−7.44
DepressionT2 → SavingsT2	−0.081 ***	0.008	−10.34

The significance level in the table is marked as: *** *p* ≤ 0.001.

**Table 5:** Robustness Test Results for Depression and Savings.

Variables	2013			2015			2018		
	Model 1	Model 2	Model 3	Model 1	Model 2	Model 3	Model 1	Model 2	Model 3
Savings	−0.345 ***	−0.314 ***	−0.291 ***	−0.517 ***	−0.490 ***	−0.460 ***	−0.513 ***	−0.488 ***	−0.465 ***
Age		0.025	0.022		0.016	0.018		0.005	0.012
Gender		1.451 ***	1.733 ***		1.389 ***	1.452 ***		1.883 ***	1.958 ***
Work Status			−0.431			−0.626 **			−0.666 **
Education level			−0.62 *			0.006			−0.121
Marital status			0.015			−0.088			−0.063
Constant			6.803 ***			8.567 ***			9.771 ***
Sample size	2194	2194	1708	2145	2145	2143	2465	2465	2465
Adjusted R-squared	0.019	0.038	0.045	0.025	0.039	0.042	0.041	0.065	0.068

The significance level in the table is marked as: *** *p* ≤ 0.001, ** *p* ≤ 0.01, * *p* ≤ 0.05.

## Data Availability

The data presented in this study are openly available in China Health and Retirement Longitudinal Study (CHARLS) at https://charls.pku.edu.cn/. For full access to the data, please contact the author Xinping Zhang to get it (xpzhang602@hust.edu.cn).
